# 

**Published:** 1886-12

**Authors:** 


CONTENTS.

Vol. 33 No. 12

PAGE.

Prophesies and Disclosures in Dreams.....321
Cancer.....326
Self-Cure.....327
Comparative Longivity of Men and Women.....328
The Cure of Disease.....329
Oxygen—Its Agency in Therapeutics.....331
Nasal Hemorrhage.....334
Treatment of Erythematous Lupus &c.....335
Ingluvin.....335
Water in Food.....337
Pasteur's Laboratory.....337
Book Notices.....338
Household.....339
Miscellaneous.....341

PAGE.

INDEX TO ADVERTISEMENTS OF HALF A PAGE AND OVER.

Fellows' Hypo-Phosphites..... I
Lactated Food..... II
Celerina..... III
Lactopeptine..... IV
Castoria..... IV
Horsford's Acid Phosphate..... V
Caulocorea..... V
Bovinine..... VI
Vaginal Injecting and Suction Syringe..... X
Medicated Suppositories..... XI
Dr. Carter Moffats' Ammoniaphone..... XII
Dr. Buckland's Scotch Oats Essence..... 2d page cover
Perforated Paper..... 3d " "
Compound Oxygen..... 4th " "

